# Effects of Taurine on Gut Microbiota Homeostasis: An Evaluation Based on Two Models of Gut Dysbiosis

**DOI:** 10.3390/biomedicines11041048

**Published:** 2023-03-29

**Authors:** Weike Qian, Mingyang Li, Leilei Yu, Fengwei Tian, Jianxin Zhao, Qixiao Zhai

**Affiliations:** 1 State Key Laboratory of Food Science and Technology, Jiangnan University, Wuxi 214122, China; 2 School of Food Science and Technology, Jiangnan University, Wuxi 214122, China

**Keywords:** taurine, gut microbiota, dysbiosis, antibiontic, *Citrobacter rodentium*

## Abstract

Taurine, an abundant free amino acid, plays multiple roles in the body, including bile acid conjugation, osmoregulation, oxidative stress, and inflammation prevention. Although the relationship between taurine and the gut has been briefly described, the effects of taurine on the reconstitution of intestinal flora homeostasis under conditions of gut dysbiosis and underlying mechanisms remain unclear. This study examined the effects of taurine on the intestinal flora and homeostasis of healthy mice and mice with dysbiosis caused by antibiotic treatment and pathogenic bacterial infections. The results showed that taurine supplementation could significantly regulate intestinal microflora, alter fecal bile acid composition, reverse the decrease in *Lactobacillus* abundance, boost intestinal immunity in response to antibiotic exposure, resist colonization by *Citrobacter rodentium*, and enhance the diversity of flora during infection. Our results indicate that taurine has the potential to shape the gut microbiota of mice and positively affect the restoration of intestinal homeostasis. Thus, taurine can be utilized as a targeted regulator to re-establish a normal microenvironment and to treat or prevent gut dysbiosis.

## 1. Introduction

Taurine (2-aminoethanesulfonic acid) is a structurally simple and abundant non-protein sulfur-amino acid in mammals that is mainly derived from the diet [1]. As a conditionally essential amino acid, taurine is widely found in the brain, heart, skeletal muscle, retina, and other tissues. Based on existing studies, we found that taurine exerts several specific physiological and pharmacological effects, such as anti-inflammatory, antioxidant, membrane stabilization, osmoregulation, cytoprotective effects, and ion transport modulation.

The major sources of taurine are endogenous synthesis and diet. The liver has been reported to be the main site of taurine biosynthesis, with two distinct pathways in which cysteine dioxygenase (CDO) and cysteine sulfate decarboxylase (CSAD) play important roles [2,3]. Since the vast majority of organisms have a low capacity for taurine biosynthesis, the level of taurine in the body is maintained by dietary intake and, consequently, the circulating level of taurine is significantly affected by diet [4]. Taurine derived from the diet is absorbed by the small intestine and subsequently transported to the liver, where it is combined with bile acids to produce conjugated bile acids that are stored in the gallbladder. These taurine-carrying bile salts are released into the duodenum after a meal and deconjugated by intestinal microorganisms, whereby taurine is released [5]. Intestinal microbes possess the ability to metabolize taurine, and this metabolic function is widely distributed [6].

Taurine has been shown to have an impact on the intestinal environment. In aged *Drosophila*, taurine supplementation inhibits intestinal stem cell hyperproliferation, attenuates age-related hyperplasia in the *Drosophila* intestine, and restrains a decline in intestinal function [7]. Dietary taurine has been reported to alleviate experimental colitis induced by dextran sulfate sodium (DSS) [8] and trinitrobenzene sulfonic acid (TNBS) [9] in mice and retard weight loss, colon shortening, and myeloperoxidase (MPO) activity elevation. Furthermore, in an imaging-based quantitative high-throughput screening to identify the host and microbiotal regulators of intestinal barrier function, taurine was identified as a new “barrier stabilizer” that blocks tight junction (TJ) disruption and the exacerbation of inflammation induced by putrescine. [10]. The relationship between taurine and intestinal microbes has gained attention in recent years. In healthy mice, the dietary addition of taurine can alter the intestinal microecology and affect its metabolism, increasing the production of short-chain fatty acids (SCFAs) [11]. One of the major differential metabolites in the serum of patients with gestational diabetes mellitus and neonatal meconium is taurine, suggesting that taurine may be associated with the neonatal gut microbiota [12]. In immunosuppressed mice, taurine supplementation has a positive effect on intestinal homeostasis by reversing the reduction in the Lachnospiraceae and Ruminococcaceae groups and improving immune cell numbers in Payer’s patches [13]. A recent report described how taurine supplementation can train the gut microbiota, leading to the expansion of taurine-utilizing taxa and functional remodeling of the microbiota. These microbes can convert taurine to ammonia, acetate, and sulfide, of which the latter inhibits the aerobic respiration of pathogenic bacteria, thereby enhancing resistance to colonization [14].

Therefore, we speculated that taurine might affect the structure of the microflora when intestinal homeostasis is disrupted and play a specific role in intestinal homeostasis and functional reconstruction. However, there is a lack of research involving these areas, and it is unknown whether taurine could be further involved in reestablishing intestinal homeostasis via gut microbiota in the context of dysbiosis. Here, we constructed two models of intestinal dysbiosis to observe the effects of taurine on the intestinal flora. In this study, taurine supplementation altered the intestinal flora and affected bile acid metabolism in healthy mice. In antibiotic-treated mice, taurine exerted a pronounced flora-modulating effect, contributing to the recovery of gut dysbiosis and eliminating the disturbance of the immune response to antibiotics. Finally, in pathogen-infected mice, taurine in the intestine trained the gut flora and enhanced the resistance to pathogens.

## 2. Materials and Methods

### 2.1. Animals and Treatments

Specific-pathogen-free (SPF) C57BL/6J mice (6 weeks old, 20–22 g) were obtained from Beijing Vital River Laboratory Animal Technology Co. Ltd. (Beijing, China). The mice were housed in a 12 h day and night cycle environment with controlled temperature (22 ± 1 °C) and humidity (50 ± 10%) and were fed an autoclaved standard diet (12.9% energy from fat, 63.9% from carbohydrates, and 23.2% from protein) for the duration of their lives. All animal experiments were approved by the Jiangsu Institute of Parasitic Diseases (permission number: IACU C-JIPD-2021152).

**Figure 1:**
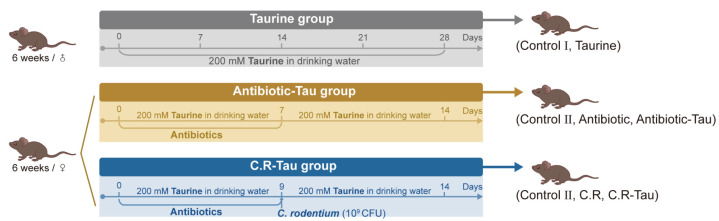
Animal experimental design.

After one week of acclimation, the mice were divided into seven treatment groups according to the experimental design. As shown in Figure 1, the treatment groups were as follows: (1) Control I group (n = 8): normal drinking water for 28 days; (2) Taurine group (n = 8): 200 mM of taurine in drinking water for 28 days; (3) Control II group (n = 10): normal drinking water for 14 days, and 0.2 mL sterile saline was administered by oral gavage on days 1 to 7; (4) Antibiotic group (n = 10): normal drinking water for 14 days, and 0.2 mL antibiotic cocktail (ampicillin, metronidazole, vancomycin, and neomycin, each 25 mg/mL; Sigma-Aldrich, Los Angeles, CA, USA) [15] was administered by oral gavage on days 1 to 7; (5) Antibiotic-Tau group (n = 10): 200 mM of taurine in drinking water for 14 days, and 0.2 mL antibiotic cocktail was administered by oral gavage on days 1 to 7; (6) C.R group (n = 10): normal drinking water for 14 days; antibiotic cocktail was administered by oral gavage on days 1 to 7 and mice were orally inoculated with *Citrobacter rodentium* (5.6 × 10^9^ CFU diluted in 200 μL PBS) on the 9th day (7) C.R-Tau group (n = 10): 200 mM of taurine in drinking water for 14 days; antibiotic cocktail was administered by gavage on days 1 to 7 and mice were orally inoculated with *C. rodentium* on the 9th day.

### 2.2. Microbiome Analysis

Total microbial DNA was extracted from fecal samples using a FastDNA Spin Kit for Feces (MP Biomedicals Ltd., Santa Ana, CA, USA). Afterward, the V3-V4 hypervariable region of the 16S rRNA gene was PCR-amplified using the universal bacterial primers 341F and 806R. After performing 2.0% agarose gel electrophoresis, the PCR products were recovered and purified using a DNA Gel/PCR Purification Miniprep Kit (Beiwo Meditech Co., Ltd., Hangzhou, China). The Illumina MiSeq platform (Illumina, Inc., San Diego, CA, USA) was employed to perform library sequencing. Quality filtering and demultiplexing were executed using the DADA2 package from Qiime2. Subsequently, open reference Amplicon sequence variants (ASVs) were employed to assign the reads. The Silva Bacterial Database was utilized for sequence alignment [16]. We analyzed diversity with Qiime2 using the Qiime diversity core plugin. We used the R (v.4.2.1) vegan, ape, ggpubr, and ggplot2 libraries for analysis and visualization and the ‘Aldex2’ package in R to further compute significant differential microbial information [17].

### 2.3. GroEL Gene-Based Analysis of Lactobacillus Species

To quantify *Lactobacillus* spp. in feces, the *GroEL* genes of *Lactobacillus* were further PCR-amplified using *Lactobacillus*-specific primers: Lac groEL F(5′-GCYGGTGCWAACCCNGTTGG-3′)/Lac groEL R(5′-AANGTNCCVCGVATCTTGTT-3′) [18]. After using 2.0% agarose gel electrophoresis, the purified DNA was sequenced by 2 × 300-bp paired-end Illumina sequencing. The DNA amplicons library was made with TruSeq DNA LT Sample Preparation Kit (Illumina, San Diego, CA, USA) and sequenced by the MiSeq Illumina platform with the MiSeq v3 Reagent Kit (600 cycles) according to the manufacturer’s instructions.

### 2.4. Bile Acid Measurements

We quantified the absolute abundance of targeted bile acids using a liquid chromatography-tandem mass spectrometry-based method [19]. In brief, the lyophilized stool sample (approximately 50 mg) was weighed, and methanol was added for crushing and homogenization. The supernatant was filtered through a 0.22-μm membrane and stored in sample vials for LC-MS analysis.

Quantitative measurement was performed using a UPLC-Q Exactive system (UPLC: UltiMate 3000; column: ACQUITY UPLC^®^ HSS T3 (1.8 µm, 2.1 × 100 mm)). The A phase (ultrapure water containing 1 mM ammonium acetate) and B phase (methanol containing 1 mM ammonium acetate) were used for elution. The detailed gradient elution conditions are shown in Supplementary Materials Table S1.

### 2.5. Quantification of Cytokines in Colon

Collected colonic tissues were accurately weighed to 100 mg and disrupted in cold saline using a high-throughput tissue disrupter. An ELISA DuoSet kit (R&D Systems China Co., Ltd., Shanghai, China) was used to determine the levels of IL-6, IL-17, and TNF-α [20].

### 2.6. Determination of CFU

Fecal samples from different time points after infection were collected in pre-weighed 1.5 mL tubes. The samples were serially diluted in 10 mL sterile saline, and each dilution was plated on LB agar containing nalidixic acid (50 μg/mL). Colony counts were performed after 24 h of incubation at 37 °C [14].

### 2.7. Statistical Analysis

All data are presented as the means ± SEM and were analyzed using GraphPad Prism 9.4.1 (GraphPad Software, San Diego, CA, USA). Unpaired two-tailed Student’s *t*-tests were used to compare data from multiple groups. When more than two groups were analyzed, significance was calculated using a one-way analysis of variance (ANOVA) followed by Tukey’s multiple comparison test. Differences were considered statistically significant at *p* < 0.05.

## 3. Results

### 3.1. Taurine Supplementation Regulated Gut Microbiota Composition in Healthy Mice

**Figure 2:**
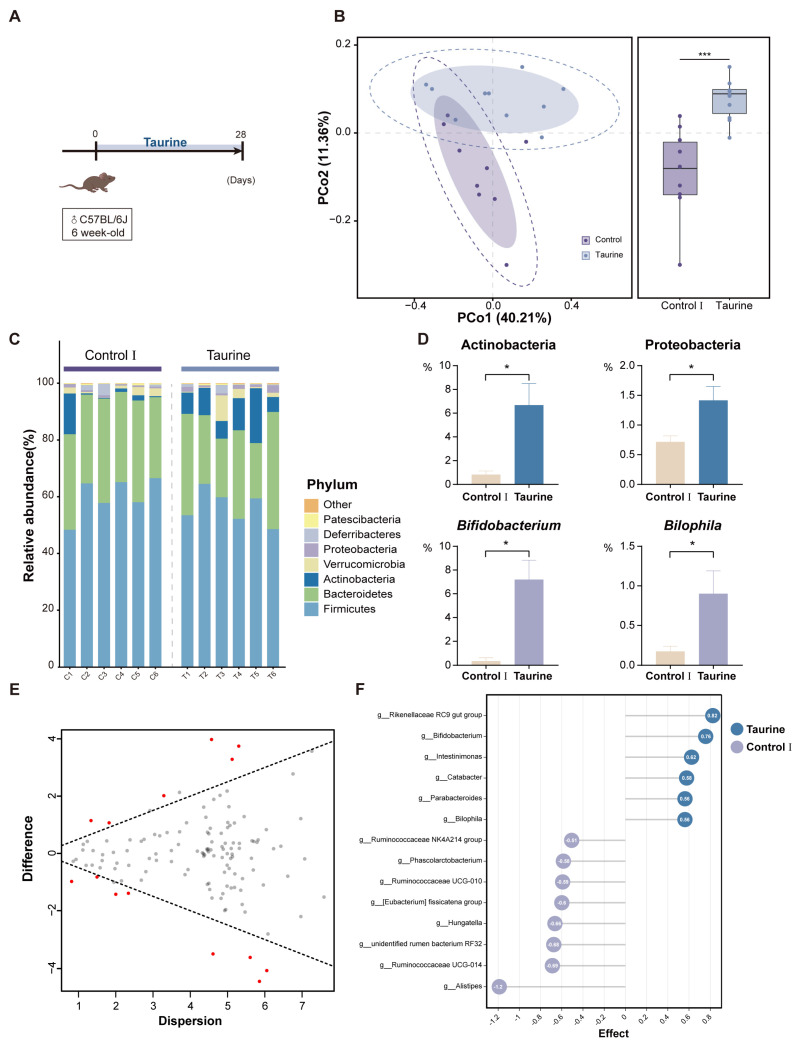
Taurine supplementation regulates the composition of gut microbiota in healthy mice. (**A**) Schematic diagram of the experimental design. (**B**) The Bray−Curtis−based principal coordinate analysis (PCoA) of gut microbiota. The boundary of the dotted ellipse: 95% confidence interval (CI); the shadow ellipse: 80% CI. Box plot center line: median; error bars: minimum to maximum. (**C**) Relative abundance of fecal microbiota at the phylum level. (**D**) Relative abundances of significantly altered phyla (top panel) and genus (bottom panel). (**E**) Effect plot between Taurine and Control Ⅰ group based on Aldex2. Features that are statistically significant are in red and those that are non−significant are in grey. (**F**) Aldex2 analysis of differences in the microbial taxa (Effect size > 0.5). Data are presented as the means ± SEM (n = 8). * *p* < 0.05 and *** *p* < 0.001 by unpaired two−tailed Student’s *t*−test.

Taurine was administered at a concentration of 200 mM in drinking water to healthy mice first in order to determine the effects of taurine supplementation on gut microbiota. After 16s rRNA sequencing of the collected fecal samples, it was found that the composition of the fecal gut microbiota was regulated by taurine supplementation. The Bray–Curtis distance-based principal coordinates analysis (PCoA) of the Control I and Taurine groups distinguished them into separate clusters. In particular, there were significant differences in PCoA2, indicating that dietary taurine supplementation altered the gut microbiota composition in healthy mice (Figure 2B). Nevertheless, α-diversity, including the Shannon and evenness index, did not differ between the two groups.

Cluster analysis at the phylum level showed that there was no significant change in the basic phylum composition of the gut microbiota after taurine supplementation (Figure 2C); however, the relative abundance of both Actinobacteria and Proteobacteria was elevated compared to that in the Control I group (Figure 2D). Among the two phyla with elevated levels, *Bifidobacterium* and *Bilophila* were the most representative genera enriched in the Taurine group (Figure 2D). Differentially abundant fecal bacterial taxa in the two groups were identified by Aldex2, and 14 discriminative features, which were mainly included in Lachnospiraceae, Bifidobacteriaceae, and Ruminococcaceae, were identified as biomarkers (Figure 2E).

### 3.2. Taurine Supplementation Affected Bile Acid Concentration and Composition in Healthy Mice

**Figure 3:**
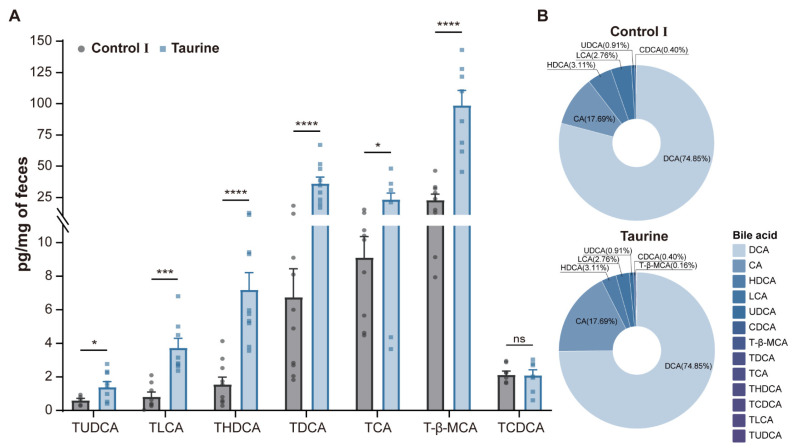
Changes in the bile acid metabolism after taurine supplementation. (**A**) Relative levels of bile acid species. (**B**) Composition of bile acids in feces. Data are presented as the means ± SEM (n = 8). * *p* < 0.05, *** *p* < 0.001, **** *p* < 0.0001, and *p* > 0.05 = not significant (ns) by unpaired two-tailed Student’s *t*-test.

Taurine supplementation resulted in a decrease in the total fecal bile acid content and a change in composition (Table 1). Levels of the majority of taurine-conjugated bile acids, including tauroursodeoxycholic acid (TUDCA), taurolithocholic acid (TLCA), taurohyodeoxycholic acid (THDCA), taurodeoxycholic acid (TDCA), taurocholic acid (TCA), and tauro-β-muricholic acid (T-β-MCA), increased significantly at different concentrations (Figure 3A), whereas the lithocholic acid (LCA) content decreased significantly. However, the taurodeoxycholic acid (TCDCA), chenodeoxycholic acid (CDCA), deoxycholic acid (DCA), ursodeoxycholic acid (UDCA), hyodeoxycholic acid (HDCA), dehydrocholic acid (DHCA), and cholic acid (CA) contents did not change significantly. The composition of bile acids was similar in both groups of mice, with no significant changes (Figure 3B).

### 3.3. Taurine Supplementation Regulated Gut Microbiota Composition and Recovery of Lactobacillus in Antibiotic-Treated Mice

**Figure 4:**
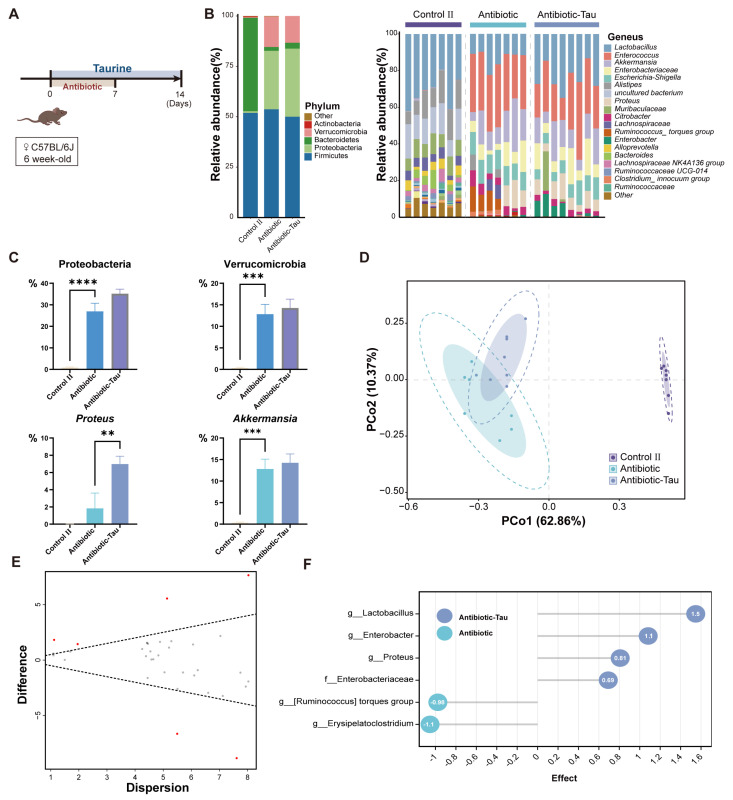
Taurine supplementation regulates the composition of gut microbiota in antibiotic−treated mice. (**A**) Schematic diagram of the experimental design. (**B**) Relative abundance of fecal microbiota at the phylum and genus (**C**) levels. (**D**) The Bray−Curtis−based principal coordinate analysis (PCoA) of gut microbiota. The boundary of the dotted ellipse: 95% confidence interval (CI), shadow ellipse: 80% CI. (**E**) Effect plot between the Antibiotic−Tau and Antibiotic group based on Aldex2. Features that are statistically significant are in red and those that are non−significant are in grey. (**F**) Aldex2 analysis of differences in the microbial taxa (Effect size > 0.5) Data are presented as the means ± SEM ** *p* < 0.01, *** *p* < 0.001, **** *p* < 0.0001, and *p* > 0.05 = not significant (ns, may not be indicated), by one−way ANOVA followed by Tukey’s test.

In the antibiotic treatment experiment, we found that antibiotic treatment could significantly change the structure and composition of intestinal flora, resulting in intestinal dysbiosis (Figure 4B). Cluster analysis at the phylum level showed that almost all phyla underwent changes in abundance after antibiotic treatment as well as taurine supplementation, with the most pronounced changes involving the relative abundance of Proteobacteria and Verrucomicrobia (Figure 4C). We applied PCoA analysis to different groups of samples at Bray–Curtis distances and showed that taurine supplementation significantly affected the composition of the gut microbiota, dividing the antibiotic and Antibiotic-Tau groups into different clusters (Figure 4D). It is noteworthy that taxa including *Lactobacillus*, Enterobacter, and Proteus were enriched in antibiotic-treated mice supplemented with taurine, whereas the other two taxa were enriched in the Antibiotic group (Figure 4E,F).

**Figure 5:**
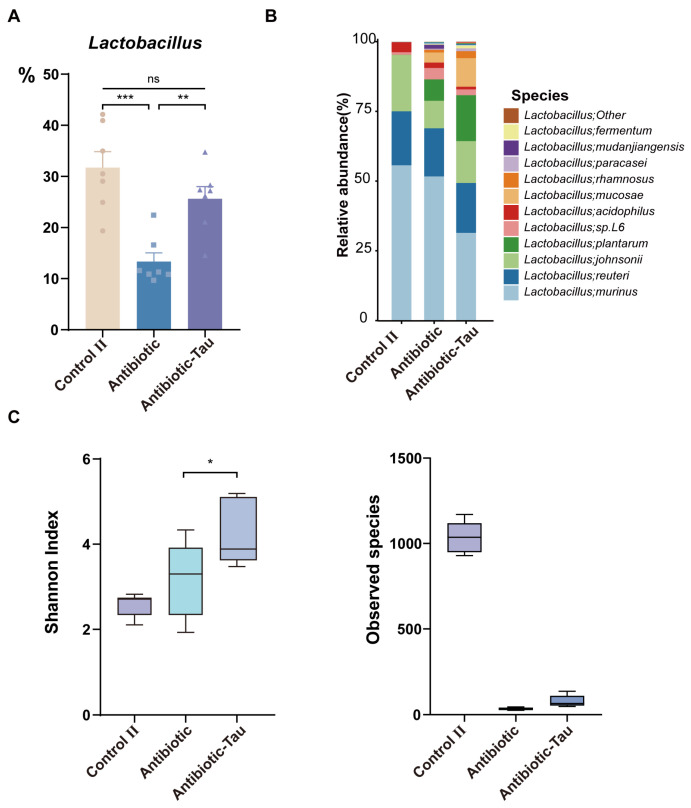
Effect of taurine supplementation on Lactobacillus during antibiotic treatment and recovery periods. (**A**) Relative abundance of *Lactobacillus* after 7 days of antibiotic exposure (Day 14). (**B**) Relative abundance of *Lactobacillus* at the species level. (**C**) Lactobacillus α-diversity during antibiotic treatment. Data are presented as the means ± SEM * *p* < 0.05, ** *p* < 0.01, *** *p* < 0.001, and *p* > 0.05 = not significant (ns, may not be indicated), by one-way ANOVA followed by Tukey’s test.

After 7 days of antibiotic exposure (day 14), we discovered that the relative abundance of *Lactobacillus* in the Antibiotic-Tau cohort was significantly higher than that in the Antibiotic group and was comparable to that in the Control II group (Figure 5A). Species-level DNA sequencing of *Lactobacillus* indicated that antibiotic treatment can significantly disrupt the structure of *Lactobacillus*, resulting in a significant reduction in observed species but an increase in the Shannon index. Furthermore, taurine supplementation can partially increase observed species and expand the difference in the Shannon index (Figure 5B,C).

### 3.4. Taurine Supplementation Boosted Immunity in Antibiotic-Treated Mice

**Figure 6:**
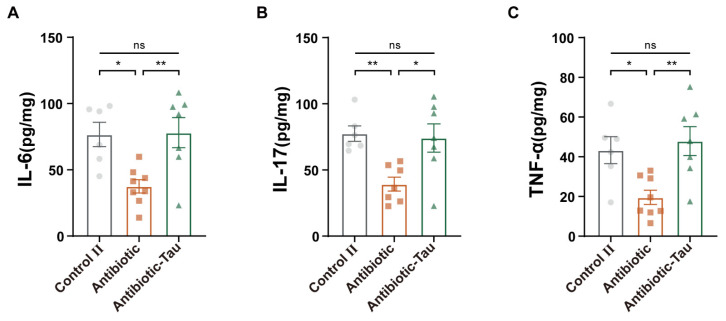
Effect of taurine on cytokines in antibiotic-treated mice. (**A**) IL-6, (**B**) IL-17, and (**C**) TNF-α. Results are presented as the means ± SEM (n = 6–8). * *p* < 0.05, ** *p* < 0.01, and *p* > 0.05 = not significant (ns), by one-way ANOVA followed by Tukey’s test.

We further measured cytokine levels in colonic tissues to assess the impact of taurine on intestinal immunity. After antibiotic treatment, IL-6, IL-17, and TNF-α concentrations were significantly reduced in mice, while those in the Antibiotic-Tau group were maintained at levels comparable to the Control II group (Figure 6). Taurine supplementation eliminated the negative effects of antibiotics on host immunity.

### 3.5. Taurine Supplementation Enhanced Colonization Resistance in Mice Infected with C. rodentium

**Figure 7:**
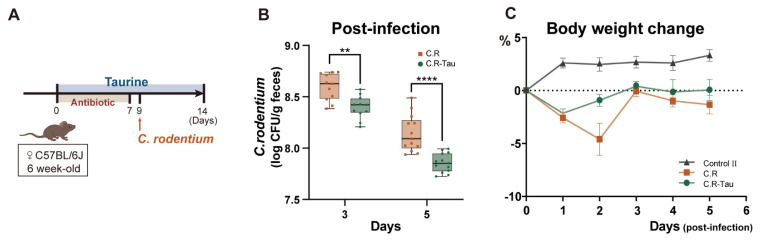
Taurine supplementation enhances colonization resistance. (**A**) Schematic diagram of the experimental design. (**B**) The fecal burden of *C. rodentium* 3− and 5−days post−infection. (**C**) Weight change from baseline (weight at 1 day before infection). Data are presented as the means ± SEM. ** *p* < 0.01 and **** *p* < 0.0001 by unpaired two-tailed Student’s *t*−test.

Following gavage infection with *C. rodentium*, mice were weighed daily and the CFUs of pathogenic bacteria were determined on days 3 and 5 post-infection. Compared to the C.R-Tau group, mice in the C.R group showed a higher fecal pathogen burden (Figure 7B). Weight loss was observed in every *C. rodentium*-infected mouse, but taurine supplementation reduced the weight loss caused by pathogenic infection (Figure 7C). These findings demonstrated that taurine supplementation enhances pathogenic resistance.

### 3.6. Taurine Supplementation Regulated the Diversity and Composition of Gut Microbiota in Mice Infected with C. rodentium

**Figure 8:**
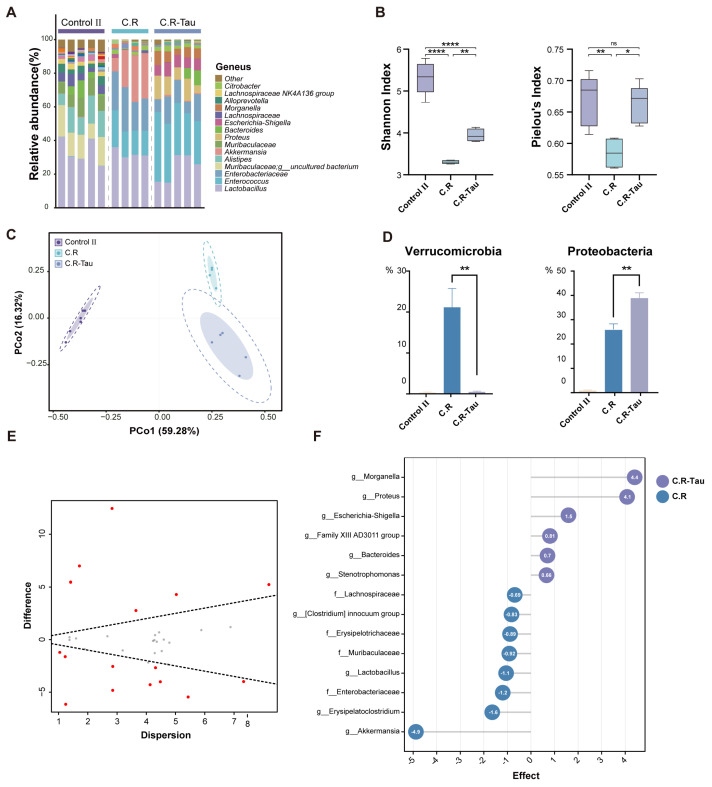
Taurine supplementation regulates the composition of gut microbiota in *C. rodentium*−infected mice. (**A**) The relative abundance of fecal microbiota at the genus level. (**B**) α-diversity of the microbiota after infection. (**C**) The Bray−Curtis based PCoA of gut microbiota. The boundary of the dotted ellipse: 95% confidence interval (CI), shadow ellipse: 80% CI. (**D**) Relative abundances of significantly changed phyla and genera. (**E**) Effect plot between the C.R−Tau and C.R group based on Aldex2. Features that are statistically significant are in red and those that are non−significant are in grey. (**F**) Aldex2 analysis of differences in the microbial taxa (Effect size > 0.5) Data are presented as the means ± SEM * *p* < 0.05, ** *p* < 0.01, **** *p* < 0.0001, and *p* > 0.05 = not significant (ns) by one-way ANOVA followed by Tukey’s test.

To investigate the effect of taurine on gut dysbiosis caused by *C. rodentium* infection, *16S* rRNA sequencing of the fecal samples was performed (Figure 8A). Based on the ASVs classification, we conducted a comparative analysis of α- and β-diversity between groups. The results showed that the C.R-Tau group displayed a higher Shannon index and evenness index than the C.R group, which were comparable to those of the Control II group (Figure 8B). We applied PCoA to the Bray–Curtis distances of samples from different groups. The microbiota profiles of both C.R and C.R-Tau mice clustered separately from those of the Control II group; the microbiota after taurine supplementation was distinct from that of the C.R group (Figure 8C).

Cluster analysis at the phylum level showed that almost all phyla underwent changes in abundance after *C. rodentium* infection as well as taurine supplementation, with the most pronounced changes involving the relative abundance of Proteobacteria and Verrucomicrobia (Figure 8D). The abundance of Proteobacteria was highest in the C.R-Tau group. We used Aldex2 analysis to identify biomarkers between the different groups as well as key genera after infection and taurine intervention. The Aldex2 results revealed that there were differences between the C. R and C.R-Tau groups, and 15 of them, including *Proteus*, *Morganella*, and *Bacteroides*, were enriched in the C.R-Tau group (Figure 8E,F).

## 4. Discussion

A balanced gut microbiota performs crucial physiological functions and prevents dysbiosis. The role of amino acids in maintaining intestinal homeostasis has garnered increasing interest in recent years. It has been demonstrated that a variety of amino acids are beneficial for intestinal barrier function [21]. The intestinal microbiota can use amino acids to produce metabolites in catabolic pathways, and host-microbe interactions are mediated by these microbiota-derived metabolites. The amino acid most frequently reported in studies related to intestinal flora is tryptophan (Trp), which is directly metabolized by microorganisms into indole, tryptamine, and indole derivatives. These metabolites can act as ligands to activate relevant receptors, improve gut barrier integrity, and relieve inflammation [22,23]. Gut microbes are both producers and utilizers of branched-chain amino acids (BCAAs; valine, isoleucine, and leucine) and can regulate BCAA levels in the gut. BCAA mixtures can promote an increase in beneficial bacterial genera, including *Akkermansia* and *Bifidobacterium*, in mice [24]. The sulfur-containing amino acid cysteine is metabolized by intestinal microorganisms to produce hydrogen sulfide (H_2_S), which colonocytes use as an energy source to provide ATP at low concentrations while inhibiting mitochondrial respiration and SCFA oxidation at higher concentrations. However, despite the well-documented effects of certain amino acids on gut microbiota composition, few studies have examined the impact of taurine supplementation.

To explore the effects of taurine on intestinal flora and metabolism, taurine was added to the drinking water of mice. As expected, PCoA analysis revealed that the gut microbiota structure was significantly impacted by taurine supplementation, although α-diversity was not obviously affected in healthy mice. The abundance of a number of specific intestinal bacteria was increased after taurine supplementation. Another result of this experiment may explain this change in flora. Taurine supplementation also induced changes in bile acid metabolism. The content and proportion of taurine-conjugated bile acids significantly increased as a result of taurine supplementation, as demonstrated by the measurement of fecal bile acids using LC-MS. The ratio of tauroconjugates to glycoconjugates may change upon taurine supplementation [25], and the taurine conjugation of bile acids has been seen to increase severalfold [26], which is consistent with our results. Bile acids are known to be generated in the liver, where they are conjugated to glycine or taurine and secreted into the intestine with the diet. Approximately 95% of these bile acids are reabsorbed in the small intestine, with only a few escaping enterohepatic circulation and reaching the colon [5]. The gut microbiota primarily uses bile salt hydrolase (BSH) deconjugation and 7-dehydroxylation processes to transform primary bile acids into secondary bile acids. BSH enzymes are prerequisites for this transformation process and are present in a variety of microorganisms. However, 7α-dehydroxylation is performed by a relatively limited set of bacteria that express the bile acid-inducible (bai) operon, including Lachnospiraceae and Ruminococcaceae [27]. In the intestinal environment, these two families are widely distributed and associated with the maintenance of intestinal health. This increase in Lachnospiraceae and Ruminococcaceae after taurine supplementation is also consistent with the findings of a previous study [13]. The released taurine is metabolized and utilized in the intestine. *Bilophila*, a strictly anaerobic, sulfite-reducing bacterium may use taurine as a substrate for its growth. However, this alteration in Proteobacteria is not entirely consistent with the findings of Yu et al.’s research [11] and may be caused by various taurine supplement sources or dosages. *Bifidobacterium* abundance differed between the two groups, and its enrichment in the Taurine group further demonstrated the influence of taurine supplementation on intestinal homeostasis. *Bifidobacterium* not only possesses BSH [28] but also contains taurine aminotransferase, which catalyzes the first step of taurine catabolism [29]. After taurine supplementation, these specific bacteria can contribute to the re-establishment of the gut microbial network.

Antibiotic treatment is one of the strongest inducers of intestinal dysbiosis. It can lead to dysbiosis of the intestinal tract, which is generally characterized by the loss of diversity, decrease in or loss of the abundance of important taxa, and the consequent effects on their metabolic capacity [30]. In several studies, the recovery phase has been identified as a crucial step in restoring gut microbiota balance [31]. Therefore, we designed this study to determine whether oral taurine supplementation could protect gut microbiota from antibiotic exposure and reverse its effects. We observed that the intestinal flora of mice were greatly altered by antibiotic administration, with a sharp decrease in diversity, whereas the relative abundance of Bacteroides declined significantly and that of Proteobacteria and Verrucomicrobia increased. Consistent with previous reports [32,33], the relative abundance of *Akkermansia* increased significantly after antibiotic treatment. This may be the result of an increase in mucus degradation following antibiotic therapy [34] which creates a more favorable habitat for *Akkermansia*. Moreover, *Akkermansia* was resistant to vancomycin and metronidazole, which were used in the experiments here [32]. In a total parenteral nutrition (TPN) study, intestinal mucosal thickness and villus height improved with taurine [35]. Taurine increases the number and function of goblet cells to improve the intestinal mucosal barrier during nematode infection [36], which also occurs when lipopolysaccharide (LPS) induces acute intestinal mucosal inflammation [37]. Unlike *Akkermansia*, *Lactobacillus* decreased among the flora after recovery from antibiotic treatment, and taurine supplementation reversed this effect. We utilized PCR primers specific to *Lactobacillus* to analyze species-level differences, and the α-diversity of *Lactobacillus* in the Antibiotic-Tau group was found to be significantly higher than that of the Antibiotic group at day 7 after the end of antibiotic exposure. Taurine exhibited the ability to increase the *Lactobacillus* observed species during the antibiotic administration period and enhance *Lactobacillus* abundance during the recovery period. Taurine and bile acids are conjugated, changing the intestinal environment, and *Lactobacillus* is rich in BSH, which can provide a growth advantage in such environments. This increase in *Lactobacillus* may be valuable in restoring antibiotic-induced disruption; as reported by Shi et al. [38], *Lactobacillus* strains restored microbial communities more effectively than natural recovery. Therefore, taurine may diminish the adverse effects of antibiotics.

Another finding was that the levels of intestinal cytokines were reduced and immunity was disrupted in antibiotic-treated mice, and that this effect could be reversed by taurine supplementation. The gut microbiota is considered to be of utmost importance in the development and maintenance of the intestinal immune system. In contrast, under antibiotic intervention, the signaling of microorganisms and their metabolites to the intestinal mucosa and peripheral organs is reduced [31], and immunoglobulin levels, immune cell numbers, and the expression and production of immune factors such as TNF-α, IFN-γ [39], IL-17 [40], and IL-6 [41] are thus affected. In addition, antibiotic treatment disrupts inflammasome-dependent cytokine activation [42]. The role of taurine in promoting immunity, in general, has been recognized [43]. In leukocytes, taurine reaches up to 50 mM and acts as an effective cytoprotective agent that increases their cell number and enhances their function [44]. Recently, it was discovered that taurine activates NLRP6, restoring the inflammasome-antimicrobial peptide axis and ameliorating colitis via intestinal microbes [45]. In the present study too, taurine exerted a protective effect on the immune system in antibiotic experiments, maintaining the abundance of various cytokines at levels comparable to those in the Control II group. Taurine could assist antibiotic-treated mice in maintaining intestinal homeostasis by balancing the intestinal flora, improving the abundance of beneficial bacteria, and enhancing immunological function.

We chose pathogenic bacterial infections as another model of gut dysbiosis. Bacterial infections are recognized as one of the major threats to human health, and the gastrointestinal (GI) tract is one of the primary sites of exposure to pathogens. Infections can threaten the homeostatic relationship with flora and lead to significant changes in the microflora, a phenomenon known as dysbiosis [46]. Antibiotics are the most commonly used clinical treatment for bacterial infections. However, apart from the aforementioned intestinal damage, antibiotic therapy usually leads to an increase in antibiotic-resistant pathogens [31]. In our study, mice were infected with *C. rodentium*, a natural mouse pathogen homologous to enterohemorrhagic *Escherichia coli* (EHEC) [15]. During infection, *C. rodentium* can disrupt the strict anaerobic environment of the intestinal tract and proliferate via aerobic respiratory metabolism. Predictably, infected mice exhibited a decrease in body weight, and taurine supplementation attenuated this effect. Beginning at day 3 post-infection, the *C. rodentium* fecal burden in the taurine-supplemented group was significantly lower than that in the non-supplemented group. Mice in the C.R-Tau group exhibited enhanced resistance to *C. rodentium* compared with those in the Control II group. Previous reports have shown that pathogens can reduce commensal diversity [47], and a similar phenomenon was observed in this study. However, taurine supplementation significantly enhanced the Shannon and evenness index of intestinal flora, reversing the decrease in α-diversity caused by *C. rodentium* infection. It is known that by competing for resources and activating an immune response, gut microbiota can resist pathogen invasion and colonization; therefore, the increased α-diversity of flora may contribute to this function. The bacteria enriched by taurine were mainly Proteobacteria, which have a functional taurine metabolic pathway. At high concentrations, taurine-derived sulfide can inhibit the activity of cytochrome oxidases [48], and pathogens are restricted from accessing non-fermentable substrates through aerobic respiration. Taurine can modify the intestinal environment and promote colonization resistance by promoting the proliferation of bacteria with probiotic effects or by being used as a substrate by intestinal microorganisms that metabolize taurine to sulfides.

## 5. Conclusions

In our experiments, taurine was administered to mice in three different physiological states. The experimental results showed that taurine regulated the intestinal microbiota and, as we hypothesized, the effect of the taurine intervention was better under intestinal instability conditions than in the healthy state. Taurine in the colon is microbially relied upon. Free taurine can be utilized as an energy source and substrate, which in turn nourishes the corresponding microorganisms. Taurine is an important signaling molecule in the intestine that regulates immune responses and enhances immunity. Sulfides produced by taurine metabolism, especially H_2_S, can inhibit the aerobic respiration of pathogens, prevent the expansion of pathogens, and enhance the resistance of host pathogenic bacteria. Taurine plays a positive role in intestinal instability, providing new insights into the taurine-mediated establishment of intestinal homeostasis and offering a possibility for the development of functional food formulations that target intestinal flora regulation.

## Figures and Tables

**Table 1:** Fecal bile acid concentration of mice after taurine supplementation.

Bile Acids	Molecular Formula	Control Ⅰ (pg/mg)	Taurine (pg/mg)	*p*-Value
TLCA	C_26_H_45_NO_5_S	0.84 ± 0.24	3.75 ± 0.51	0.0003 ***
TDCA	C_26_H_45_NO_6_S	6.78 ± 1.58	36.37 ± 4.69	<0.0001 ****
THDCA	C_26_H_45_NO_6_S	1.59 ± 0.39	7.22 ± 0.95	<0.0001 ****
TUDCA	C_26_H_45_NO_6_S	0.64 ± 0.08	1.42 ± 0.28	0.0218 *
T-β-MCA	C_26_H_45_NO_7_S	23.27 ± 4.09	99.09 ± 10.84	<0.0001 ****
TCA	C_26_H_45_NO_7_S	9.13 ± 1.16	23.60 ± 4.67	0.0148 *
TCDCA	C_26_H_45_NO_6_S	3.53 ± 0.88	3.85 ± 1.20	0.8402
LCA	C_24_H_40_O_3_	3187.10 ± 579.74	1687.12 ± 232.76	0.0351 *
CDCA	C_24_H_40_O_4_	218.27 ± 48.82	241.55 ± 68.35	0.8002
DCA	C_24_H_40_O_4_	58,850.10 ± 9636.26	45,730.93 ± 4574.82	0.2585
UDCA	C_24_H_40_O_4_	552.15 ± 144.52	555.15 ± 139.47	0.9889
HDCA	C_24_H_40_O_4_	3801.19 ± 837.05	1902.31 ± 203.96	0.0509
CA	C_24_H_40_O_5_	7746.34 ± 2424.70	10,806.87 ± 2805.21	0.4503

The concentration of bile acid is presented as the mean ±SEM. *p*-values (*t*-test) were obtained compared to the Control group with the same bile acid content. * *p* < 0.05, *** *p* < 0.001, and **** *p* < 0.0001.

## Data Availability

Not applicable.

## Supplementary Materials

The following supporting information can be downloaded at: https://www.mdpi.com/article/10.3390/biomedicines11041048/s1, Table S1: The elution gradient; Table S2. Aldex2 analysis of differences in the microbial taxa between Taurine and Control Ⅰ group; Table S3. Aldex2 analysis of differences in the microbial taxa between Antibiotic-Tau and Antibiotic group; Table S4. Aldex2 analysis of differences in the microbial taxa between C.R-Tau and C.R group.
