# Supplementary material for: Effects of Taurine on Gut Microbiota Homeostasis: An Evaluation Based on Two Models of Gut Dysbiosis
Source: Biomedicines. 2023 Mar 29;11(4):1048. doi: 10.3390/biomedicines11041048 (PMC10135931; doi:10.3390/biomedicines11041048)
Supplement: Supplementary file 1 [file biomedicines-11-01048-s001.zip › biomedicines-2272059-supplementary.pdf]

Supporting Information for

Effects of taurine on gut microbiota homeostasis: An evaluation based on two models of gut dysbiosis

Weike Qian[1,2], Mingyang Li[1,2], Leilei Yu[1,2], Fengwei Tian[1,2], Jianxin Zhao[1,2], Qixiao Zhai[1,2,*]

1 State Key Laboratory of Food Science and Technology, Jiangnan University, Wuxi, Jiangsu 214122 P. R China

2 School of Food Science and Technology, Jiangnan University, Wuxi, Jiangsu 214122, China.

*Corresponding Author: Qixiao Zhai

E-mail addresses: zhaiqixiao@jiangnan.edu.cn

The file includes:

Table S1 to S4

Table S1. The elution gradient

Time (min)	A	B
0	80%	20%
0-6	80-40%	20-60%
6-25	0%	100%
25-26	0%	100%
26-28	0-50%	100-50%
28-30	50-80%	50-20%
30-32	80%	20%

Flow rate of 0.3 mL/min, column temperature was maintained at 30℃.

Table S2. Aldex2 analysis of differences in the microbial taxa between Taurine and Control I group. Positive values indicate higher abundances in Taurine group, while negative values correspond to higher abundances in Control I group.

**Name**	**Effect**	**Name**	**Effect**
Rikenellaceae RC9 gut group	0.82418	*Alistipes*	-1.18895
Bifidobacterium	0.757043	*Ruminococcaceae UCG-014*	-0.68847
Intestinimonas	0.623993	*unidentified rumen bacterium RF32*	-0.67556
Catabacter	0.577016	*Hungatella*	-0.66442
Parabacteroides	0.561738	*[Eubacterium] fissicatena group*	-0.59929
Bilophila	0.561361	*Ruminococcaceae UCG-010*	-0.59161
Butyricimonas	0.499737	*Phascolarctobacterium*	-0.58475
ASF356	0.443217	*Ruminococcaceae NK4A214 group*	-0.50662

Table S3. Aldex2 analysis of differences in the microbial taxa between Antibiotic-Tau and Antibiotic group. Positive values indicate higher abundances in Antibiotic-Tau, while negative values correspond to higher abundances in Antibiotic group.

**Name**	**Effect**	**Name**	**Effect**
Lactobacillus	1.547984	*Erysipelatoclostridium*	-1.05332
Enterobacter	1.082583	*[Ruminococcus] torques group*	-0.97657
Proteus	0.808426	*Alistipes*	-0.42612
f__Enterobacteriaceae	0.691757	*uncultured bacterium*	-0.42288
Enterococcus	0.492112		
Akkermansia	0.434226		

Table S4. Aldex2 analysis of differences in the microbial taxa between C.R-Tau and C.R group. Positive values indicate higher abundances in C.R-Tau, while negative values correspond to higher abundances in C.R group.

Name	Effect	Name	Effect
Morganella	4.401302	*Akkermansia*	-4.88299
Proteus	4.07961	*Erysipelatoclostridium*	-1.64422
Escherichia-Shigella	1.539724	f__Enterobacteriaceae	-1.19842
Family XIII AD3011 group	0.805517	*Lactobacillus*	-1.05555
Bacteroides	0.701172	f__Muribaculaceae	-0.91965
Stenotrophomonas	0.658571	f__Erysipelotrichaceae;g__uncultured	-0.88847
		[Clostridium] innocuum group	-0.82702
		f__Lachnospiraceae	-0.68934
